# Human Platelet Lysate Media Supplement Supports Lentiviral Transduction and Expansion of Human T Lymphocytes While Maintaining Memory Phenotype

**DOI:** 10.1155/2019/3616120

**Published:** 2019-09-04

**Authors:** Emanuele Canestrari, Hayley R. Steidinger, Brianna McSwain, Steven J. Charlebois, Christina Tenenhaus Dann

**Affiliations:** Cook Regentec, Indianapolis, Indiana, USA

## Abstract

Immune cell therapy has emerged as a promising approach to treat malignancies that were up until recently only treated on a palliative basis. Chimeric antigen receptor- (CAR-) modified T lymphocytes (T cells) in particular have proven to be very effective for certain hematological malignancies. The production of CAR T cells usually involves viral transduction and *ex vivo* culture of T cells. The aim of this study was to explore the use of human platelet lysate (HPL) compared to two commonly used supplements, human AB serum (ABS) and fetal bovine serum (FBS), for modified T cell production. For studying transduction, activated T cells were transduced with lentivirus to deliver GFP transgenes with three different promoters. Transduction efficiency (percent GFP) was similar among the supplements, and a modest increase in the transgene product (mean fluorescence intensity) was observed when HPL was used as a supplement compared to ABS. To study the effect of supplements on expansion, peripheral blood mononuclear cells (PBMCs) were activated and expanded in the presence of interleukin 2 (IL2) for fourteen days. T cell expansions using HPL and ABS were comparable and slightly less than the expansion obtained with FBS. Interestingly, cells expanded in media supplemented with HPL showed a higher percentage of T cells with a central memory phenotype compared to those expanded in ABS or FBS. Protein profiling revealed that the phenotypic differences may be explained by elevated levels of several cytokines in HPL, including IL7. The results suggest that the use of HPL as a cell culture supplement during the production of modified T cells is a reasonable alternative to ABS. Furthermore, the use of HPL may enhance *in vivo* performance of the final product by enriching for central memory T cells that are associated with long-term persistence following adoptive transfer.

## 1. Introduction

Cellular adoptive immunotherapy with *ex vivo*-modified T lymphocytes (T cells) has emerged as a promising therapeutic strategy to treat various cancer and autoimmune diseases [1, 2]. T cells engineered to express chimeric antigen receptors (CARs) have elicited high rates of clinical response particularly against pediatric acute lymphoblastic leukemia, and efforts to target solid tumors are promising and ongoing [3, 4]. The manufacture of CAR T cell therapies typically begins with the collection of mononuclear cells via leukapheresis, followed by genetic modification with viral vectors and *ex vivo* expansion of the modified cells [5].

Although the factors determining clinical efficacy of CAR T cell therapy are not entirely understood, the field continues to look for biological features of infused cells that lead to better clinical response. Mounting evidence supports the vital role that naïve (T_N_) and central memory (T_CM_) subtypes play in long-term persistence and clinical efficacy compared with unselected or highly differentiated T cells [6–9]. Additionally, recent studies have generated a model wherein a balance between CD4^+^ and CD8^+^ cells is desirable in immunotherapy transfusion products [7, 10, 11].

As more immune cell therapies move into the clinic, there is an increasing demand for high quality media supplements that are not supply-limited. The use of fetal bovine serum (FBS) as a supplement for T cell culture carries a risk of pathogen transmission as well as xenoimmunization against bovine antigens [12, 13]. Human AB serum (ABS), currently the most common media supplement for T cell culture in a clinical context, has supply limitations and may not be sufficient to meet the expected demand for immunotherapies [14]. Significant effort has been put into the development of serum-free media formulations to overcome these limitations as well as the increasing regulatory requirements associated with the use of serum in the manufacture of cells for adoptive therapies [15]. While some improvement of *ex vivo* T cell growth has been achieved, many serum-free formulations result in inferior growth kinetics compared to serum-supplemented media [16–18].

Human platelet lysate (HPL) obtained from transfusable donor platelets is widely recognized as a valuable alternative to both FBS and human ABS for the production of clinical cell therapies [19, 20]. While HPL is a common supplement for the culture of mesenchymal stromal cells, including for use in cell therapy, and recent reports describe its application for culturing macrophages and monocyte-derived dendritic cells, to our knowledge, the use of HPL for culturing T cells has not been published [21, 22]. HPL is available from some blood banks and commercial suppliers and recently has become available in a pathogen-reduced grade (e.g., nLiven PR™), which is designed to meet the more stringent regulatory requirements for the mitigated risk of transmission of residual virus from a human-derived product.

The aim of the current study was to evaluate the use of HPL as a nonxenogeneic culture media supplement for processes required during the production of T cells for adoptive T cell therapy. Therefore, we systematically compared the effects of HPL, FBS, and ABS on lentiviral transduction, *ex vivo* expansion, and memory phenotype of human T cells. Our data suggest that HPL not only supports *ex vivo* transduction and expansion of T cells but also increases the central memory phenotype compared to T cells cultured with ABS. Altogether, the data support the idea that HPL may be considered as a reasonable alternative to ABS, thereby expanding options for cell culture supplements as demand increases.

## 2. Methods

### 2.1. Lentiviral Transduction

All transductions were performed on pan-T cells, which were purchased from STEMCELL Technologies or purified in-house from peripheral blood mononuclear cells (PBMCs) (Indiana Blood Center) from healthy donors using the EasySep™ Human T Cell Isolation Kit (STEMCELL Technologies). PBMCs were isolated from the blood using standard procedures of density gradient centrifugation. Cells were cultured in AIM V medium (Thermo Fisher Scientific) containing 100 IU/mL of human recombinant interleukin 2 (IL2) (STEMCELL Technologies), or where indicated, 5 ng/mL IL15 or 5 ng/mL IL7 together with 5 ng/mL IL15 (PeproTech), and 5% (*v*/*v*) of either FBS (Thermo Fisher Scientific, Corning), heat-inactivated human ABS (Sigma-Aldrich, Valley Biomedical), or HPL (nLiven PR™, pathogen-reduced HPL, Cook Regentec).

The HPL used in this study was produced using the manufacturing process or in some cases, a scaled down version, for the commercially available nLiven PR™. Expired platelet units were sourced from FDA-registered and AABB-certified blood banks and must have been acceptable for allogeneic transfusion prior to expiration. Donors underwent thorough screening and testing prior to donation to reduce the risk of transfusion-transmitted infections. Platelets were collected into bags containing an ACD-A anticoagulant and frozen within forty-eight hours of expiration. For the production of each lot of HPL, approximately 100 donor platelet units were thawed, pooled, electron beam (E-beam) irradiated, and manipulated to rupture the platelet membrane. E-beam irradiation was selected as a pathogen reduction method because it has the capacity to reduce potential transfusion-transmitted infections while maintaining the product's ability to perform as a cell culture media supplement [23]. The resultant lysate was then filtered and storage vessels aseptically filled. No heparin was used during the production of HPL or its use as a cell culture supplement. Standard assays, such as osmolality and total protein content, were performed on each lot to ensure lot-to-lot consistency. In addition, cytokines were quantified by ELISA (R&D Systems) and results for a subset of factors relevant to T cells are shown in Supplementary [Supplementary-material supplementary-material-1].

Phosphoglycerate kinase-green fluorescent protein (PGK-GFP) and elongation factor 1 alpha-GFP (EF1A-GFP) lentiviruses were obtained from Cellomics, and cytomegalovirus-GFP (CMV-GFP) lentivirus was obtained from Indiana University Vector Production Facility (Indianapolis, IN). Pilot experiments were performed to attain a relative infectious titer for each viral vector. A low multiplicity of infection (MOI) (<1) was chosen to allow the detection of potential differences between conditions, and MOIs used for each of the three vectors were not designed to be comparable because of differing inherent activity of each promoter. Prior to transduction, cells were thawed and activated with the ImmunoCult™ Human CD3/CD28 T Cell Activator (STEMCELL Technologies), then cultured for four days to give the cells time to recover from thawing and become activated. Cells were counted on day four, and transductions were set up in a 96-well plate with 100,000 cells per well in media containing 2.14 × 10^6^ mol/L hexadimethrine bromide (polybrene; Sigma) and lentivirus. Although polybrene is unlikely to be used in a clinical setting, it is a transduction enhancer commonly used for research. Analysis was performed using BD Accuri C6 and C6-Plus instruments and software (BD Biosciences). Analysis was at two days after transduction, to evaluate the effect of supplement on early aspects of transduction such as viral entry, and seven days after transduction when reporter expression is thought to be derived from stable integration [24]. Cells were gated based on forward and side scatter plots, and GFP^+^ cells were defined based on comparison to untransduced cells. Arithmetic mean fluorescence intensity (MFI) of GFP^+^ cells was obtained and reported as a ratio for ABS or HPL relative to FBS for each donor or as an arbitrary unit in the experiments not involving FBS.

### 2.2. T Cell Expansion

For expansion experiments, cryopreserved PBMCs (STEMCELL Technologies) from three donors were thawed, activated, and cultured with the same reagents as described above. PBMCs were seeded in a 24-well plate at a density of 1 × 10^6^ cells/mL. Supplements were used at 5% (*v*/*v*), and three lots of each supplement were tested for each donor. In the case of expansion in IL15 and/or IL7, a single lot of HPL and ABS was compared and three donors were used that were different from the donors used for expansion in IL2. Cells were counted and cell density adjusted to 0.5 − 1 × 10^6^ cells/mL by adding fresh media starting on the fourth day, then every 2-3 days. Viability was quantified using trypan blue exclusion. Cell counts from the three lots of each supplement were considered as technical replicates and averaged. Cells were analyzed by immunostaining and flow cytometry on day 14 of culture.

### 2.3. Immunostaining and Flow Cytometry

Prior to staining, cells were blocked using an FcR blocking reagent (Miltenyi Biotec). Cell phenotype was analyzed by immunostaining. Monoclonal antibodies were from BD Biosciences except where indicated. Antibodies used included (1) anti-CD3-allophycocyanin (APC) (Miltenyi Biotec, clone REA613), CD62L-PE (clone SK11), and CD56-fluorescein isothiocyanate (FITC) (BioLegend, clone 5.1H11); (2) CD4-R-phycoerythrin-cyanine 7 (clone RPA-T4), CD8-R-phycoerythrin (PE) (clone RPA-T8), CD197 (CCR7)-FITC (clone 150503), and CD45RO-APC (clone UCHL1); or (3) CD62L-Alexa Fluor 647 (clone SK11). Fluorescence minus one (FMO) controls and appropriate isotype controls were performed. An AbC Total Antibody Compensation Bead Kit (Thermo Fisher Scientific) was used to define compensation settings. Analysis was performed using BD Accuri C6 instruments and software (BD Biosciences).

### 2.4. Cytokine Analysis

Samples from three different lots of ABS and HPL were sent to RayBiotech Inc. for quantitative analysis with the Quantibody® Human Cytokine Antibody Array 640. This array system utilized the multiplexed sandwich ELISA-based technology to quantitatively measure the concentration of 640 human cytokines. IL7 concentration was independently measured using the Human IL-7 SimpleStep ELISA Kit (Abcam) according to the manufacturer's instructions. Absorbance was measured at 450 nm with a DTX880 multimode detector (Beckman Coulter).

### 2.5. Gene Ontology Analysis

The biological annotations of the differentially enriched proteins were assessed by Gene Ontology (GO) analysis using DAVID bioinformatics resources version 6.8 (https://david.ncifcrf.gov/home.jsp) using the GOTERM_BP_DIRET category. Proteins with a fold change (FC) ≥ 10 times and *P* value ≤ 0.01 were included in the analysis. GO terms with a false discovery rate (FDR) less than 0.05 were considered statistically significant.

### 2.6. Statistical Analysis

Statistical analyses were performed using GraphPad Prism 7 (GraphPad) software. Cell analysis data were obtained using three donors. Protein analysis data were from at least three lots of each supplement, as indicated. Data are from multiple independent experiments and are presented as mean ± standard deviation (SD). Differences between groups were analyzed by one-way analysis of variance (ANOVA) or multiple *t*-test. A *P* value < 0.05 was considered significant.

## 3. Results

### 3.1. HPL Enhances Transgene Expression following Lentiviral Transduction

**Figure 1:**
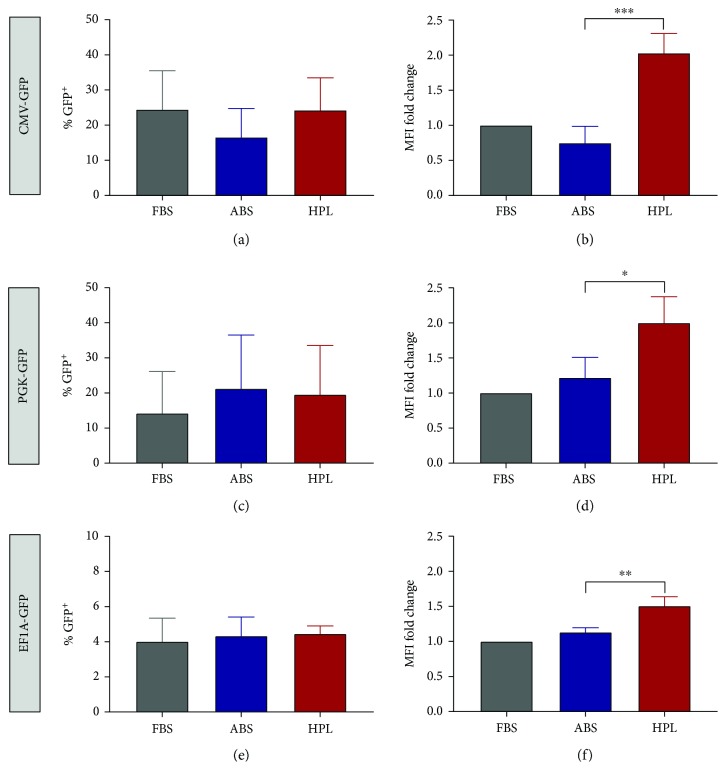
Effect of supplements on lentiviral transduction of primary T cells analyzed at day seven after transduction. (a, c, e) Percent GFP positive or (b, d, f) mean fluorescence intensity (MFI) seven days after transduction in FBS, ABS, or HPL with lentivirus to deliver CMV-GFP (a, b), PGK-GFP (c, d), or EF1A-GFP (e, f). Each graph shows mean ± SD for three donors. ^∗^*P* < 0.05, ^∗∗^*P* < 0.01, and ^∗∗∗^*P* < 0.001.

We evaluated the efficiency of genetic modification of primary T cells following activation and lentiviral transduction with a CMV-GFP reporter transgene in media containing FBS, ABS, or HPL. The overall transduction efficiency (percent GFP^+^) varied widely among the three different donors' cells that were tested, consistent with what has been observed with clinical samples (range at day 7 of 15%-37% for FBS, 10%-26% for ABS, and 14%-30% for HPL) [5]. Similar transduction efficiency was observed among the supplements tested at day seven after transduction (Figure 1(a) and Supplementary [Supplementary-material supplementary-material-1]) and at day two after transduction (Supplementary [Supplementary-material supplementary-material-1]). Interestingly, we observed an increase of transgene levels (mean fluorescence intensity or MFI) of GFP^+^ cells in HPL (2.0 ± 0.3-fold) vs. ABS (0.7 ± 0.2-fold), relative to FBS at day seven (*P* = 0.0008) (Figure 1(b)). A second set of transduction experiments was performed wherein a range of MOIs were tested using three different donors and different lots of ABS and HPL than were used in the first set of experiments. At all three MOIs tested, MFI was significantly higher in HPL than in ABS at day seven after transduction, confirming previous results. However, in this set of donors, transduction efficiency (percent GFP^+^) was significantly higher in HPL than in ABS at day 7 (Supplementary [Supplementary-material supplementary-material-1]). The discrepancy in results related to transduction efficiency may be because of the small number of donors tested in each set of experiments.

While the CMV promoter is commonly used to drive transgene expression in a fundamental research setting, the EF1A promoter is commonly used to drive CAR transgenes in T cells in a clinical setting. To test whether the effect of HPL on GFP MFI was promoter dependent, we performed a second set of experiments with T cells from three additional donors using lentiviruses to deliver EF1A-GFP, as well as another reporter commonly used in research, PGK-GFP. Like with the CMV-GFP transgene, PGK-GFP and EF1A-GFP MFI were increased compared to ABS at two (Supplementary [Supplementary-material supplementary-material-1]) and seven days (Figures 1(d) and 1(f)) after transduction using the HPL supplement. However, it should be noted that the relative increase of EF1A-GFP in HPL versus ABS was modest (<2-fold). The mechanism leading to the higher transgene expression in HPL was unclear. The observations that all three transgenes were increased in HPL suggested that it was not a promoter-specific phenomenon.

### 3.2. T Cell Expansion in FBS, ABS, and HPL

**Figure 2:**
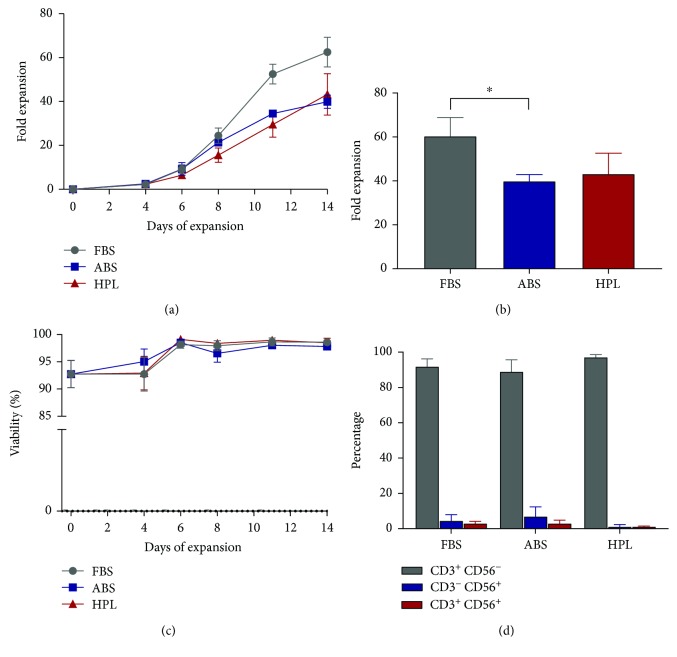
(a) Expansion kinetics of PBMCs activated with CD3/CD28 antibodies. Cells were cultured in media supplemented with 5% FBS, ABS, or HPL in the presence of 100 IU/mL IL2. (b) Total fold expansion at day 14 of culture. Mean ± SD of three donors is shown. ^∗^*P* < 0.05. (c) Mean viability of cells expanded in culture media supplemented with FBS, ABS, or HPL. (d) Percentage of lymphocyte subsets after 14 days of PBMCs cultured with different media supplements.

Cryopreserved PBMCs were used to compare the growth of T cells from three donors in three lots of each supplement in the presence of IL2. We found that T cells expanded robustly in all supplements tested, with FBS yielding the greatest fold expansion (62.5 ± 6.8-fold) whereas HPL and ABS yielded a slightly lower but similar fold expansion to each other (43.3 ± 9.4- and 40 ± 3-folds, respectively) (Figures 2(a) and 2(b)). Cell viability was higher than 90% for all the tested supplements throughout the expansion process (Figure 2(c)). We also examined the final composition of cells present after expansion. The percentage of T cells (CD3^+^CD56^−^) present in the initial cell population averaged 67.4%, and by the end of the two-week expansion, FBS and ABS showed a similar concentration of T cells, 91.9% and 89.0%, respectively, while HPL showed a slightly higher but not statistically different concentration, 97.3% (Figure 2(d) and Table 1). This increase of the T cell population with HPL-supplemented media relative to FBS and ABS was in part counterbalanced by a reduction of the CD3^−^CD56^+^ population (1.3%) compared to FBS (4.6%) and ABS (7.0%). However, these differences were not statistically significant. In conclusion, at the end of the expansion, CD3^+^CD4^+^ T cells represented most of the cells present in all three conditions, with no significant differences between the three groups (Table 1).

### 3.3. HPL Boosts T Naïve/Central Memory Markers, CCR7 and CD62L

**Figure 3:**
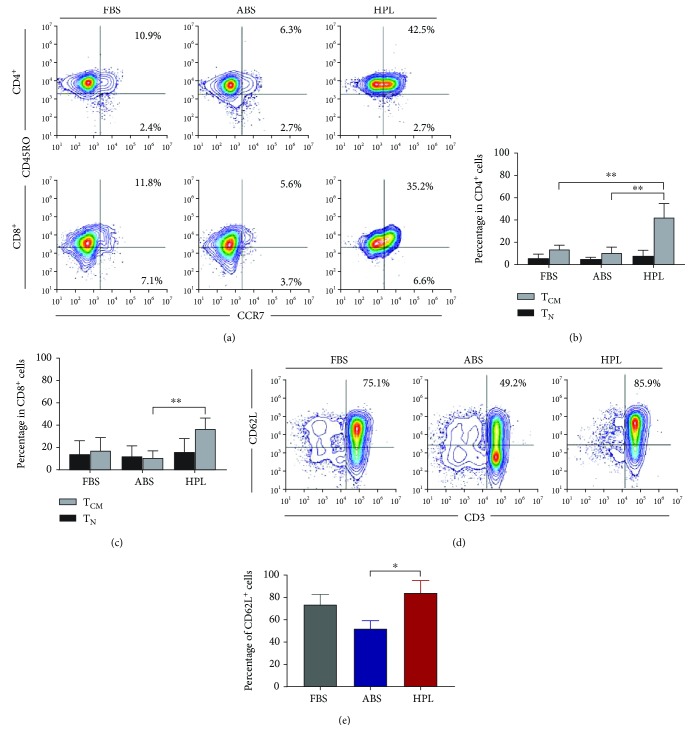
(a) Representative flow cytometry analysis of expanded T cells. The expression of CCR7 and CD45RO markers was assessed on day 14 of culture for CD4^+^- or CD8^+^-gated T cells. (b, c) Percentage of CCR7^+^/CD45RO^−^ (T_N_) and CCR7^+^/CD45RO^+^ (T_CM_) fractions in CD4^+^ and CD8^+^ T cells, respectively. Mean ± SD of three donors is shown. ^∗∗^*P* < 0.01. (d, e) Representative flow cytometry analysis and percentage of CD62L-positive T cells at day 14 of culture. Mean ± SD of three donors is shown. ^∗^*P* < 0.05.

We examined the effect of the different supplements on T cell maturation after CD3/CD28 stimulation by performing immunostaining using CD62L or a combination of CCR7 and CD45RO, to assess the subtypes of CD4^+^ and CD8^+^ T cells (Figure 3(a)). We found that the phenotype of the expanded T cells varied depending on the supplement used. T cells expanded in HPL consistently comprised a higher fraction of cells with a central memory (T_CM_) phenotype (CCR7^+^/CD45RO^+^), compared to T cells expanded in ABS or FBS (Figures 3(b) and 3(c)). The CD4^+^/CCR7^+^/CD45RO^+^ cells were 42.3% for HPL vs. 13.7% for FBS (*P* = 0.005) and 10.5% for ABS (*P* = 0.001). The CD8^+^/CCR7^+^/CD45RO^+^ cells were 36.6% for HPL vs. 17.2% for FBS (not significant) and 10.7% for ABS (*P* = 0.009). No differences were observed for the naïve (T_N_) population (CCR7^+^/CD45RO^−^) between the different groups for either CD4^+^ or CD8^+^ cells (Figures 3(b) and 3(c)). Analysis of another homing molecule associated with naïve and central memory T cells, CD62L (Figure 3(d)), further confirmed the increase of the T_N_/T_CM_ population in cells expanded with HPL with 84.3% of CD62L-positive cells compared to 52.1% for ABS (*P* = 0.01) and 73.7% for FBS (not significant) (Figure 3(e)). Altogether, the immunostaining analyses suggested that culturing in HPL could allow one to produce a T cell population that is enriched in less differentiated subtypes.

While IL2 has historically been a widely used supplement for T cell culture, recent studies have shown that a combination of IL7 and IL15 can support T cell expansion while preserving the less differentiated T_N_ phenotype [25–27]. We compared the expansion of PBMCs in ABS and HPL in the presence of IL7 plus IL15 and in the presence of IL15 alone. We found that both ABS and HPL supported robust cell growth in the presence of either IL7/IL15 or IL15 alone (Supplementary [Supplementary-material supplementary-material-1]). Fold expansion on day 14 was significantly higher with HPL than with ABS (227.3 ± 38.9-fold for HPL vs. 72.2 ± 4.4-fold for ABS in IL15 alone, *P* = 0.016; 290.3 ± 22.6-fold for HPL vs. 157.2 ± 20.2-fold for ABS in IL7/IL15, *P* = 0.011). Phenotype analysis of cells on day 14 of expansion showed a significant increase in T_CM_ and a nonsignificant increase in T_N_ in HPL (Supplementary [Supplementary-material supplementary-material-1]-[Supplementary-material supplementary-material-1]). CD62L staining was also elevated in HPL compared to ABS (Supplementary [Supplementary-material supplementary-material-1] and [Supplementary-material supplementary-material-1]). Altogether, the data further support the idea that cells cultured in HPL exhibit a less differentiated phenotype than cells cultured in ABS.

### 3.4. Protein Profile Analysis

**Figure 4:**
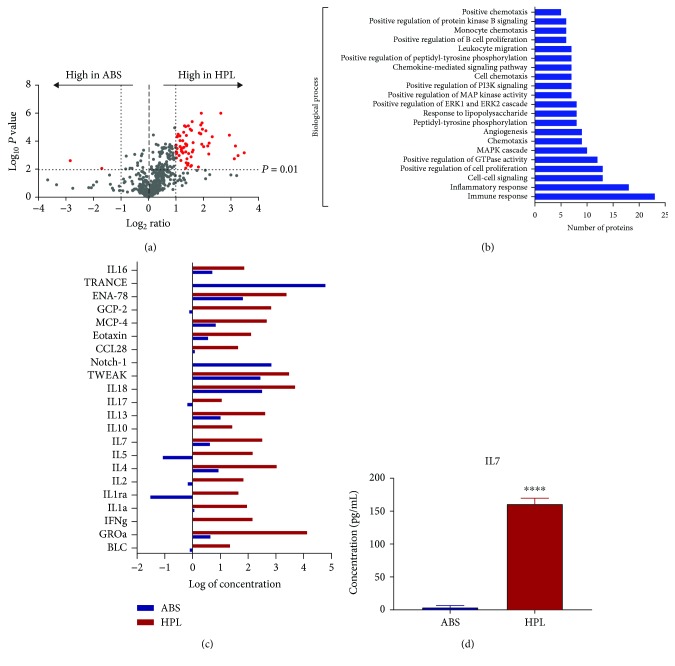
Proteomic profiling of ABS and HPL. (a) Volcano plot showing the distribution of quantified proteins, for three lots each of ABS and HPL, according to the *P* value and fold change. Red dots represent proteins with a fold change (FC) ≥ 10 and *P* value ≤ 0.01. (b) Gene Ontology analysis of 69 proteins with FC ≥ 10 and *P* ≤ 0.01. Graph shows GO terms with FDR ≤ 0.05. (c) Plot showing concentration of 22 proteins involved in the immune response biological process based on GO analysis. (d) IL7 concentration in ABS and HPL based on ELISA. Graph shows mean values ± SD of four (ABS) or five (HPL) different lots. ^∗∗∗∗^*P* < 0.0001.

In order to identify factors potentially associated with enrichment of the central memory phenotype in T cells expanded with HPL, we measured the concentrations of 640 cytokines in three lots of ABS and three lots of HPL. A protein was considered detectable and therefore included in the final analysis, when concentration values were above the limit of detection in at least two of the three analyzed lots. A total of 477 proteins were detectable in ABS compared to 544 in HPL. There were 451 proteins detectable in both ABS and HPL, 26 proteins detectable in ABS but not in HPL, and 101 proteins detectable in HPL but not in ABS. A total of 70 proteins were detectable in neither ABS nor HPL. The cytokine profiling showed a trend for an overall higher concentration of proteins in HPL compared to ABS (Figure 4(a)) in spite of the fact that the total protein concentrations of the analyzed samples were similar (HPL range 5.2–5.7 g/dL and ABS range 5.2–7.3 g/dL). Using a fold change difference ≥ 10 times and a *P* value ≤ 0.01 as a threshold, we identified a list of 69 proteins differentially enriched between ABS and HPL (Supplementary [Supplementary-material supplementary-material-1]). We next investigated the biological functions of those 69 proteins with the Gene Ontology functional annotation tool, and we identified a total of 21 terms associated with biological processes with FDR ≤ 0.05 (Figure 4(b)). Among all terms, immune response showed the highest number of associated proteins with 22 proteins and most of those proteins were at a higher concentration in HPL compared to ABS (Figure 4(c)). Among the proteins found to be enriched in HPL was IL7, which has been previously shown to be able to sustain *in vivo* expansion of central memory T cells [28]. Further analysis of IL7 levels in multiple lots of ABS and HPL consistently confirmed its enrichment in HPL (Figure 4(d)).

## 4. Discussion

With expanding investigation and commercialization of cell therapies, there is also increased demand for technology that supports production of the therapeutic cells. Establishing a method for generating a therapeutic cell product involves balancing numerous factors. One must be able to produce the cells efficiently and robustly while minimizing risk of failure and risk of introducing adventitious agents. For autologous cell therapy production, there is additional risk of failure resulting from inconsistent starting material. For cutting edge cellular therapeutics, measurable attributes that accurately predict product efficacy are typically ill-defined and awaiting discoveries to illuminate the underlying biology.

One of the key elements to producing any cellular therapeutic is the culture media. Natural blood-derived supplements are often thought to provide a rich mixture of components, albeit largely undefined, needed to support *in vitro* culture of what can be finicky starting material common in autologous cell therapy. For over half a century, FBS was the traditional supplement chosen for *in vitro* culture of cells. While generally effective for many cell types, its acceptance for the production of therapeutic cells has waned and is likely to be completely replaced as alternatives become available.

Human-derived supplements for cell culture, including ABS and HPL, have the advantage of being xeno-free yet introduce the risk of carrying human-derived pathogens. Current screening procedures applied in North America include human immunodeficiency virus (HIV), hepatitis B virus (HBV), hepatitis C virus (HCV), syphilis, and West Nile virus (WNV) screening and can contribute to minimize the risk. Nevertheless, there are some potential blood-borne infections, including nonendemic pathogens or new viral strains, for which diagnostic tests may not be available or not routinely performed by the supplier. Hence, the implementation of pathogen reduction treatments can further decrease the risk of contamination and can complement routine pathogen screening. Several pathogen reduction methods have been proven to reduce a broad range of bacteria and enveloped viruses in platelet lysate products. Some of the pathogen reduction methods used for HPL are solvent-detergent [29], amotosalen/UV-A (Intercept®, Cerus) [29, 30], short-wave UV-C illumination (Theraflex-UV®, Macopharma) [31], riboflavin/UV-A/-B (Mirasol®, Terumo BCT) [30], and gamma irradiation [32]. The efficacy of E-beam for viral inactivation of liquid matrices has also been widely addressed [23], and we have found that the use of E-beam is a feasible and effective pathogen reduction method for HPL [33].

Products sourced from donor blood material have the potential for lot-to-lot inconsistency. A way to overcome inconsistency resulting from biological variation among donors is to pool material from many donors. Although information regarding number of donors pooled for a typical lot of ABS is not always available, each lot of HPL used in this study was derived from a pool of a least 100 donors. We used a pathogen-reduced form of HPL because meeting the increasing regulatory requirements is an ongoing need for producers of cellular therapeutics.

The goal of our study was to compare the use of HPL to ABS and FBS for producing genetically modified T cells. We found that HPL supported transduction and expansion of T cells while increasing the central memory phenotype compared to T cells cultured with ABS. As with most burgeoning fields, there is little uniformity in the methods used to generate CAR T cells including the starting cell types, basal media, type of vessel/growth platform, method of T cell activation, and viral delivery approach. Differences in the methods used can and do lead to both subtle and sometimes significant differences in the outcome. The results from our study are encouraging and will require further investigation to establish whether they extend to other scenarios such as patient cells, CAR transgenes, retroviral delivery, or different culture systems.

One observation we made is that T cells transduced in HPL exhibit about 1.5- to 2-fold higher transgene expression levels (MFI) than cells in ABS. MFI was increased with all three promoters tested, multiple donors' cells, and using multiple lots of ABS and HPL. Expression of a lentiviral transgene is influenced by several aspects such as virus entry, genome integration, promoter activity, and protein translation/turnover. Additionally, T cell transduction is influenced by activation state [24, 34, 35]. Although it is unclear how culturing T cells in HPL leads to higher transgene expression, the effect was observed at the earliest reasonable time point for detecting a transgene-derived product (two days), suggesting that HPL may influence an early step such as activation, virus entry, and/or virus integration. GFP expression at two days posttransduction may in part reflect the product from unintegrated transgene (pseudotransduction), but the observation of higher MFI in HPL was sustained at seven days posttransduction suggesting that the effect was not transient [24, 36]. Increased transgene expression might generally be considered a favorable effect; however, in certain cases, including possibly CAR expression, “overactivity” may lead to inferior function [37]. Further testing for specific transgenes of interest will ultimately be necessary in the context of specific culture systems to optimize the amount of virus needed to obtain the desired expression levels and functional output.

Defining desirable attributes of a cell product like CAR T cells is a complex matter. Nonetheless, one quality that has emerged as being correlated with higher efficacy in the clinic is the presence of undifferentiated T cells (T_N_ and T_CM_) [9, 38]. Particularly, central memory and a more recently described T cell subset called stem cell memory cells are thought to exhibit dual properties of proliferative capacity and effector potential that may be associated with long-term remission [39, 40]. We found that T cell expansion in HPL, compared to ABS, leads to a statistically significant enrichment in cells with higher expression of CCR7 and CD62L, typifying the T_N_/T_CM_ populations. We have observed varying effects of HPL specifically on the T_N_ population, characterized by CCR7^+^/CD45RO^−^, and further experiments will be needed to tease out possible effects specifically on T_N_ or stem cell memory subtypes. Human platelet lysate and human serum exhibit fairly substantial differences in their composition, and it is likely that the phenotypic differences of cells cultured in each supplement may be explained by differences in the quantities of cytokines known to affect the T cell phenotype. Indeed, we demonstrated that IL7 is at significantly higher levels in HPL than in ABS. Possibly the enrichment of IL7 in HPL, alone or together with other cytokines or even nonprotein components, may contribute to the phenotype of T cells cultured in HPL. Consistent with a role for IL7 in HPL, we found that robust expansion can be obtained with HPL and the single cytokine IL15, in contrast to the more commonly used culture condition of ABS with both IL7 and IL15. Unlike IL7, IL15 and another cytokine for T cell expansion, IL21, were not consistently detected in either HPL or ABS samples in our proteomic analyses.

In addition to the T cell differentiation state, the presence of CD4^+^ T cells in infused cell products is important for effective tumor cell killing by CD8^+^ T cells. Recent studies both in murine models and in human patients showed a significant enhancement in response when a 1 : 1 mixture of CD4^+^ and CD8^+^ cells was formulated [7, 10, 11]. None of the culture conditions we tested consistently yielded the ideal ratio of equal parts CD4^+^ and CD8^+^ cells, and no significant difference was observed in the resulting CD4/CD8 ratio following expansion in the various supplements. A range of CD4/CD8 ratios obtained following the expansion of PBMCs, generally from about 30% to 70% CD4^+^ cells, have been observed by others, and it depends on several factors including the method of activation and media composition [16, 41, 42]. Nonetheless, in our study, all supplements supported the expansion of both CD4^+^ and CD8^+^ cells.

## 5. Conclusion

The variable composition and transducability of patient-derived cells creates challenges for autologous cell manufacturing. Recent emphasis on processes that produce higher quality cells and disease-free survival following infusion of lower cell doses with defined composition underscores the importance of quality over quantity [8, 11, 41]. Our data demonstrate the feasibility of using HPL as a media supplement to support the *ex vivo* transduction and expansion processes involved in producing modified T cells. Future experiments will be important for determining if the current findings may be extrapolated to the transduction of patient cells with CAR and other transgenes. Nonetheless, our results support the idea that HPL may be considered as a reasonable and potentially preferable alternative to ABS, thereby expanding options for cell culture supplements as demand increases. Finally, the availability of a pathogen-reduced HPL may increase safety and addresses the potentially increasing regulatory needs for risk mitigation.

## Figures and Tables

**Table 1:** Results of immunostaining/flow cytometry following expansion.

	Donor	CD3^+^CD4^+^ (percent)	CD3^+^CD8^+^ (percent)	CD3^+^CD56^−^(percent)	CD3^+^CD56^+^ (percent)	CD3^−^CD56^+^ (percent)
Day 0	1	48.7	18.0	63.6	2.7	12.2
	2	69.2	13.8	81.2	3.6	7.9
	3	38.8	17.6	57.6	4.0	19.5
	Mean ± SD	52.2 ± 15.5	16.5 ± 2.3	67.4 ± 12.3	3.4 ± 0.7	13.2 ± 5.9

FBS	1	66.5	21.5	91.1	3.1	5.4
	2	77.1	10.7	88.1	4.2	7.5
	3	88.0	5.9	96.6	2.0	0.9
	Mean ± SD	77.2 ± 10.8	12.7 ± 8.0	91.9 ± 4.3	3.1 ± 1.1	4.6 ± 3.4

ABS	1	61.4	23.4	86.9	3.5	9.1
	2	72.1	12.9	83.6	4.6	11.0
	3	89.7	5.6	96.5	1.2	1.0
	Mean ± SD	74.4 ± 14.3	14.0 ± 8.9	89.0 ± 6.7	3.1 ± 1.7	7.0 ± 5.3

HPL	1	80.7	11.2	95.8	1.6	2.5
	2	92.3	4.0	97.8	1.2	1.0
	3	85.2	5.8	98.3	1.2	0.3
	Mean ± SD	86.1 ± 5.8	7.0 ± 3.7	97.3 ± 1.3	1.3 ± 0.2	1.3 ± 1.1

Phenotype of cells before expansion (day 0) and after 14 days of expansion using different supplements. Data represent percentage of positive cells. FBS: fetal bovine serum; ABS: AB negative serum; HPL: human platelet lysate.

## Data Availability

The data used to support the findings of this study are available from the corresponding author upon request.

## Supplementary Materials

Supplementary Materials Supplementary Figure 1: (a) protein analysis by Biuret or Bradford assays in ten lots of HPL. Mean of the ten lots is represented by a line. (b) Cytokine analysis by ELISA of cytokines, CXCL12 (SDF1), TGFB1, and IGF1 in ten lots of HPL. Mean of the ten lots is represented by a line for each cytokine. Supplementary Figure 2: (a) representative dot plot showing gating of CMV-GFP^+^ cells (right) compared to untransduced (“NT”) cells (left). (b) Representative histogram showing the distribution of GFP fluorescence in untransduced cells (“NT”) or cells transduced with CMV-GFP lentivirus in FBS, ABS, or HPL. Supplementary Figure 3: effect of supplements on lentiviral transduction of primary T cells analyzed at day two after transduction. (a, c, e) Percent GFP positive or (b, d, f) mean fluorescence intensity (MFI) two days after transduction in FBS, ABS, or HPL with lentivirus to deliver CMV-GFP (a, b), PGK-GFP (c, d), or EF1A-GFP (e, f). Each graph shows mean ± SD for three donors. ^∗^*P* < 0.05 and ^∗∗∗^*P* < 0.001. Supplementary Figure 4: comparison of ABS and HPL supplementation during transduction of primary T cells at multiple multiplicities of infection (MOIs). (a, c) Percent GFP positive or (b, d) mean fluorescence intensity (MFI) at two days (a, b) or seven days (c, d) after transduction. Each graph shows mean ± SD for three donors. 1x is an amount of the CMV-GFP virus equivalent to that used in other figures, and 5x and 10x are 5-fold and 10-fold higher amounts of the virus. ^∗^*P* < 0.05. Supplementary Figure 5: (a) expansion kinetics of PBMCs activated with CD3/CD28 antibodies. Cells were cultured in media supplemented with 5% ABS or HPL in the presence of IL15 (a) or IL15 plus IL7 (b) at 5 ng/mL. (c–f) The expression of CCR7 and CD45RO markers was assessed on day 14 of culture for CD4^+^- or CD8^+^-gated T cells. Percentage of CCR7^+^/CD45RO^−^ (T_N_) and CCR7^+^/CD45RO^+^ (T_CM_) fractions in CD4^+^ (c, d) and CD8^+^ (e, f) T cells, respectively. Mean ± SD of three donors is shown. ^∗^*P* < 0.05 and ^∗∗∗^*P* < 0.001. (g, h) Percentage of CD62L^+^ T cells at day 14 of culture. Mean ± SD of three donors is shown. ^∗^*P* < 0.05. Supplementary Table S1: differentially enriched proteins in HPL vs. ABS.

## References

[1] June C. H., O’Connor R. S., Kawalekar O. U., Ghassemi S., Milone M. C. (2018). CAR T cell immunotherapy for human cancer. *Science*.

[2] Maldini C. R., Ellis G. I., Riley J. L. (2018). CAR T cells for infection, autoimmunity and allotransplantation. *Nature Reviews Immunology*.

[3] Gauthier J., Yakoub-Agha I. (2017). Chimeric antigen-receptor T-cell therapy for hematological malignancies and solid tumors: clinical data to date, current limitations and perspectives. *Current Research in Translational Medicine*.

[4] Geyer M. B., Brentjens R. J. (2016). Review: current clinical applications of chimeric antigen receptor (CAR) modified T cells. *Cytotherapy*.

[5] Vormittag P., Gunn R., Ghorashian S., Veraitch F. S. (2018). A guide to manufacturing CAR T cell therapies. *Current Opinion in Biotechnology*.

[6] Berger C., Jensen M. C., Lansdorp P. M., Gough M., Elliott C., Riddell S. R. (2008). Adoptive transfer of effector CD8^+^ T cells derived from central memory cells establishes persistent T cell memory in primates. *The Journal of Clinical Investigation*.

[7] Sommermeyer D., Hudecek M., Kosasih P. L. (2016). Chimeric antigen receptor-modified T cells derived from defined CD8^+^ and CD4^+^ subsets confer superior antitumor reactivity *in vivo*. *Leukemia*.

[8] Wu F., Zhang W., Shao H. (2013). Human effector T cells derived from central memory cells rather than CD8^+^ T cells modified by tumor-specific TCR gene transfer possess superior traits for adoptive immunotherapy. *Cancer Letters*.

[9] Xu Y., Zhang M., Ramos C. A. (2014). Closely related T-memory stem cells correlate with in vivo expansion of CAR.CD19-T cells and are preserved by IL-7 and IL-15. *Blood*.

[10] Moeller M., Haynes N. M., Kershaw M. H. (2005). Adoptive transfer of gene-engineered CD4^+^ helper T cells induces potent primary and secondary tumor rejection. *Blood*.

[11] Turtle C. J., Hanafi L. A., Berger C. (2016). CD19 CAR-T cells of defined CD4^+^:CD8^+^ composition in adult B cell ALL patients. *The Journal of Clinical Investigation*.

[12] Erickson G. A., Bolin S. R., Landgraf J. G. (1991). Viral contamination of fetal bovine serum used for tissue culture: risks and concerns. *Developments in Biological Standardization*.

[13] Selvaggi T. A., Walker R. E., Fleisher T. A. (1997). Development of antibodies to fetal calf serum with arthus-like reactions in human immunodeficiency virus-infected patients given syngeneic lymphocyte infusions. *Blood*.

[14] Brindley D. A., Davie N. L., Culme-Seymour E. J., Mason C., Smith D. W., Rowley J. A. (2012). Peak serum: implications of serum supply for cell therapy manufacturing. *Regenerative Medicine*.

[15] Agency, EM (2013). Guideline on the use of bovine serum in the manufacture of human biological medicinal products.

[16] Alnabhan R., Gaballa A., Mörk L. M., Mattsson J., Uhlin M., Magalhaes I. (2018). Media evaluation for production and expansion of anti-CD19 chimeric antigen receptor T cells. *Cytotherapy*.

[17] Medvec A. R., Ecker C., Kong H. (2018). Improved expansion and *in vivo* function of patient T cells by a serum-free medium. *Molecular Therapy - Methods & Clinical Development*.

[18] Sato K., Kondo M., Sakuta K. (2009). Impact of culture medium on the expansion of T cells for immunotherapy. *Cytotherapy*.

[19] Burnouf T., Strunk D., Koh M. B. C., Schallmoser K. (2016). Human platelet lysate: replacing fetal bovine serum as a gold standard for human cell propagation?. *Biomaterials*.

[20] Hemeda H., Giebel B., Wagner W. (2014). Evaluation of human platelet lysate versus fetal bovine serum for culture of mesenchymal stromal cells. *Cytotherapy*.

[21] Svajger U. (2017). Human platelet lysate is a successful alternative serum supplement for propagation of monocyte-derived dendritic cells. *Cytotherapy*.

[22] Tylek T., Schilling T., Schlegelmilch K. (2019). Platelet lysate outperforms FCS and human serum for co-culture of primary human macrophages and hMSCs. *Scientific Reports*.

[23] Nims R. W., Plavsic M. (2015). Efficacy of electron beam for viral inactivation. *Journal of Microbial & Biochemical Technology*.

[24] Costello E., Munoz M., Buetti E., Meylan P. R. A., Diggelmann H., Thali M. (2000). Gene transfer into stimulated and unstimulated T lymphocytes by HIV-1-derived lentiviral vectors. *Gene Therapy*.

[25] Alvarez-Fernández C., Escribà-Garcia L., Vidal S., Sierra J., Briones J. (2016). A short CD3/CD28 costimulation combined with IL-21 enhance the generation of human memory stem T cells for adoptive immunotherapy. *Journal of Translational Medicine*.

[26] Cieri N., Camisa B., Cocchiarella F. (2013). IL-7 and IL-15 instruct the generation of human memory stem T cells from naive precursors. *Blood*.

[27] Priesner C., Aleksandrova K., Esser R. (2016). Automated enrichment, transduction, and expansion of clinical-scale CD62L(+) T cells for manufacturing of gene therapy medicinal products. *Human Gene Therapy*.

[28] Okoye A. A., Rohankhedkar M., Konfe A. L. (2015). Effect of IL-7 therapy on naive and memory T cell homeostasis in aged rhesus macaques. *The Journal of Immunology*.

[29] Barro L., Su Y. T., Nebie O. (2019). A double‐virally‐inactivated (intercept–solvent/detergent) human platelet lysate for in vitro expansion of human mesenchymal stromal cells. *Transfusion*.

[30] Kwon S. Y., Kim I. S., Bae J. E. (2014). Pathogen inactivation efficacy of mirasol PRT system and intercept blood system for non-leucoreduced platelet-rich plasma-derived platelets suspended in plasma. *Vox Sanguinis*.

[31] Viau S., Chabrand L., Eap S. (2017). Pathogen reduction through additive-free short-wave UV light irradiation retains the optimal efficacy of human platelet lysate for the expansion of human bone marrow mesenchymal stem cells. *PLoS One*.

[32] Viau S., Eap S., Chabrand L., Lagrange A., Delorme B. (2019). Viral inactivation of human platelet lysate by gamma irradiation preserves its optimal efficiency in the expansion of human bone marrow mesenchymal stromal cells. *Transfusion*.

[33] Charlebois S., Canestrari E., Harris S. (2018). Characterization of a pathogen reduced human platelet lysate. *Cytotherapy*.

[34] Frecha C., Costa C., Negre D. (2008). Stable transduction of quiescent T cells without induction of cycle progression by a novel lentiviral vector pseudotyped with measles virus glycoproteins. *Blood*.

[35] Unutmaz D., KewalRamani V. N., Marmon S., Littman D. R. (1999). Cytokine signals are sufficient for HIV-1 infection of resting human T lymphocytes. *The Journal of Experimental Medicine*.

[36] Case S. S., Price M. A., Jordan C. T. (1999). Stable transduction of quiescent CD34^+^CD38^–^ human hematopoietic cells by HIV-1-based lentiviral vectors. *Proceedings of the National Academy of Sciences of the United States of America*.

[37] Frigault M. J., Lee J., Basil M. C. (2015). Identification of chimeric antigen receptors that mediate constitutive or inducible proliferation of T cells. *Cancer Immunology Research*.

[38] Busch D. H., Fräßle S. P., Sommermeyer D., Buchholz V. R., Riddell S. R. (2016). Role of memory T cell subsets for adoptive immunotherapy. *Seminars in Immunology*.

[39] Gattinoni L., Lugli E., Ji Y. (2011). A human memory T cell subset with stem cell-like properties. *Nature Medicine*.

[40] Klebanoff C. A., Gattinoni L., Restifo N. P. (2012). Sorting through subsets: which T-cell populations mediate highly effective adoptive immunotherapy?. *Journal of Immunotherapy*.

[41] Kaartinen T., Luostarinen A., Maliniemi P. (2017). Low interleukin-2 concentration favors generation of early memory T cells over effector phenotypes during chimeric antigen receptor T-cell expansion. *Cytotherapy*.

[42] Tumeh P. C., Koya R. C., Chodon T. (2010). The impact of ex vivo clinical grade activation protocols on human T-cell phenotype and function for the generation of genetically modified cells for adoptive cell transfer therapy. *Journal of Immunotherapy*.

