# Supplementary material for: Human Platelet Lysate Media Supplement Supports Lentiviral Transduction and Expansion of Human T Lymphocytes While Maintaining Memory Phenotype
Source: J Immunol Res. 2019 Sep 4;2019:3616120. doi: 10.1155/2019/3616120 (PMC6746159; doi:10.1155/2019/3616120)
Supplement: Supplementary Materials — Supplementary Figure 1: (a) protein analysis by Biuret or Bradford assays in ten lots of HPL. Mean of the ten lots is represented by a line. (b) Cytokine analysis by ELISA of cytokines, CXCL12 (SDF1), TGFB1, and IGF1 in ten lots of HPL. Mean of the ten lots is represented by a line for each cytokine. Supplementary Figure 2: (a) representative dot plot showing gating of CMV-GFP+ cells (right) compared to untransduced (“NT”) cells (left). (b) Representative histogram showing the distribution of GFP fluorescence in untransduced cells (“NT”) or cells transduced with CMV-GFP lentivirus in FBS, ABS, or HPL. Supplementary Figure 3: effect of supplements on lentiviral transduction of primary T cells analyzed at day two after transduction. (a, c, e) Percent GFP positive or (b, d, f) mean fluorescence intensity (MFI) two days after transduction in FBS, ABS, or HPL with lentivirus to deliver CMV-GFP (a, b), PGK-GFP (c, d), or EF1A-GFP (e, f). Each graph shows mean ± SD for three donors. ∗P < 0.05 and ∗∗∗P < 0.001. Supplementary Figure 4: comparison of ABS and HPL supplementation during transduction of primary T cells at multiple multiplicities of infection (MOIs). (a, c) Percent GFP positive or (b, d) mean fluorescence intensity (MFI) at two days (a, b) or seven days (c, d) after transduction. Each graph shows mean ± SD for three donors. 1x is an amount of the CMV-GFP virus equivalent to that used in other figures, and 5x and 10x are 5-fold and 10-fold higher amounts of the virus. ∗P < 0.05. Supplementary Figure 5: (a) expansion kinetics of PBMCs activated with CD3/CD28 antibodies. Cells were cultured in media supplemented with 5% ABS or HPL in the presence of IL15 (a) or IL15 plus IL7 (b) at 5 ng/mL. (c–f) The expression of CCR7 and CD45RO markers was assessed on day 14 of culture for CD4+- or CD8+-gated T cells. Percentage of CCR7+/CD45RO− (TN) and CCR7+/CD45RO+ (TCM) fractions in CD4+ (c, d) and CD8+ (e, f) T cells, respectively. Mean ± SD of three donors is shown. ∗P < 0.0 [file 3616120.f1.pdf]

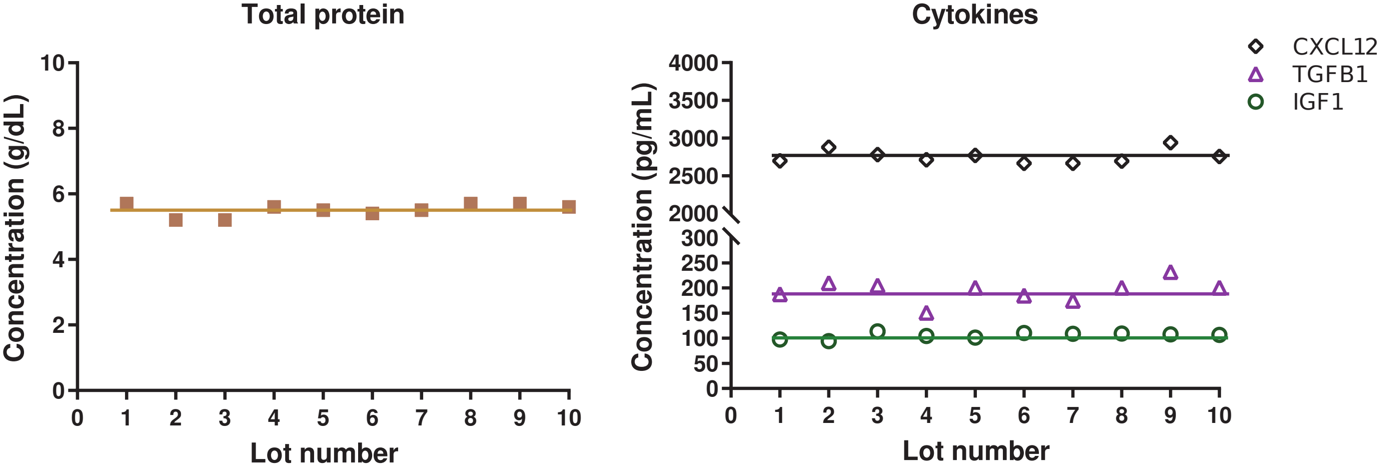

Total protein
Concentration (g/dL)
0
2
4
6
8
10
0 1 2 3 4 5 6 7 8 9 10
Lot number
Cytokines
Concentration (pg/mL)
0
50
100
150
200
250
300
2000
2500
3000
3500
4000
0 1 2 3 4 5 6 7 8 9 10
Lot number
CXCL12
TGFB1
IGF1

(a)
(b)

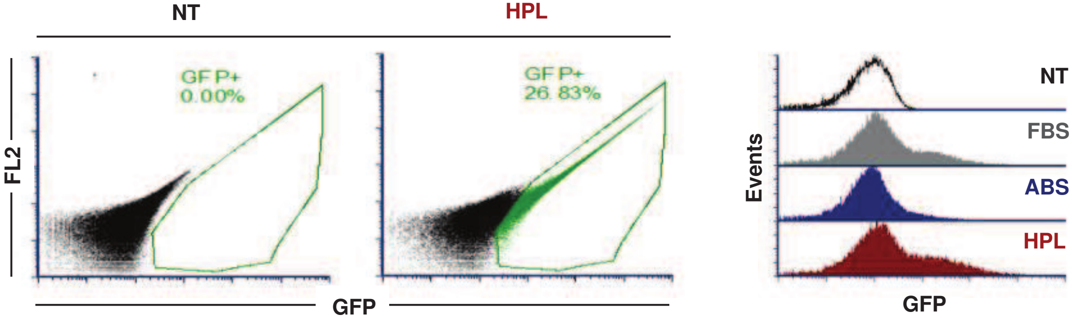

(a) (b)

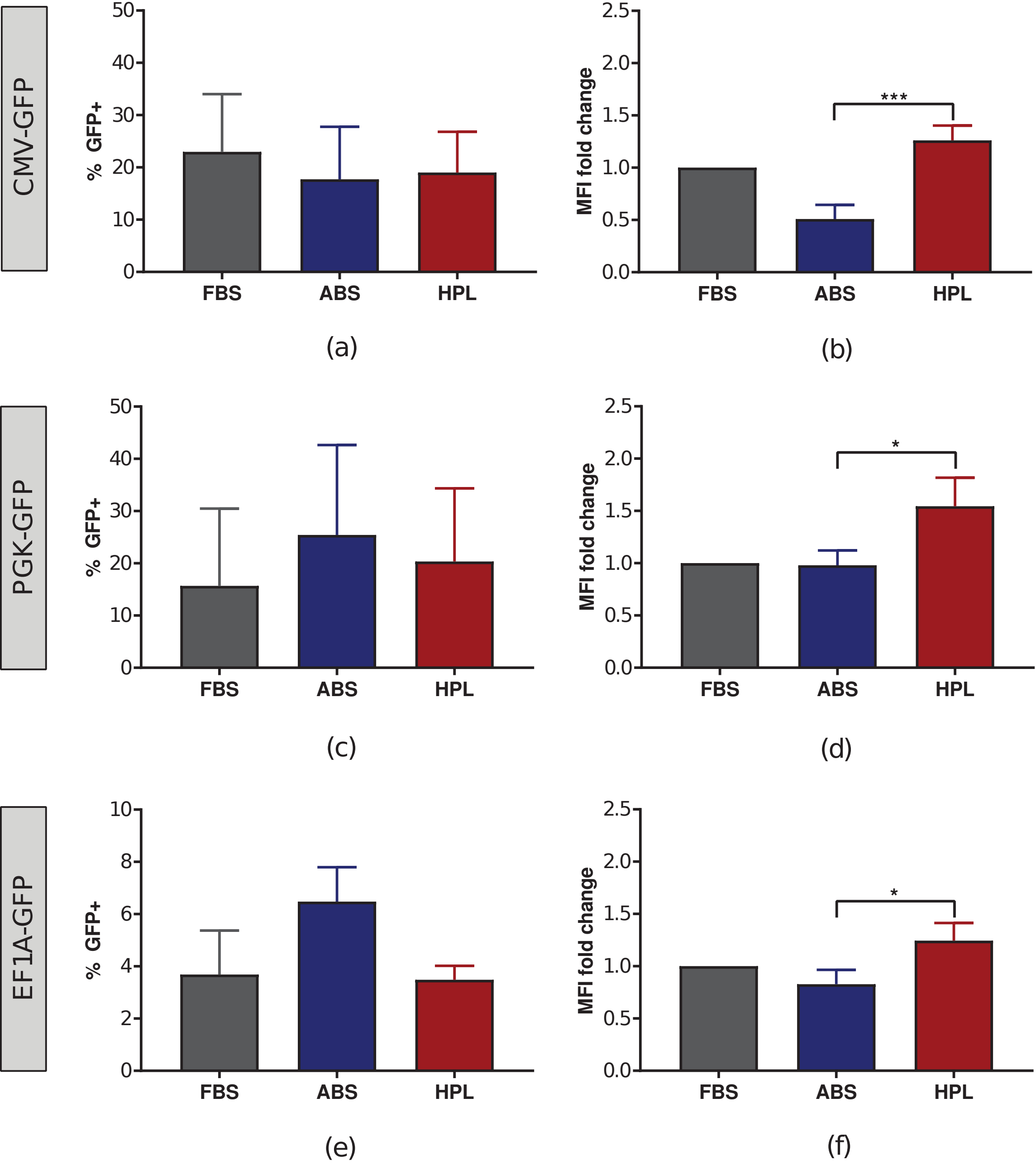
CMV-GFP
PGK-GFP
EF1A-GFP
% GFP+
MFI fold change
FBS
ABS
HPL
***
*
*
(a)
(b)
(c)
(d)
(e)
(f)

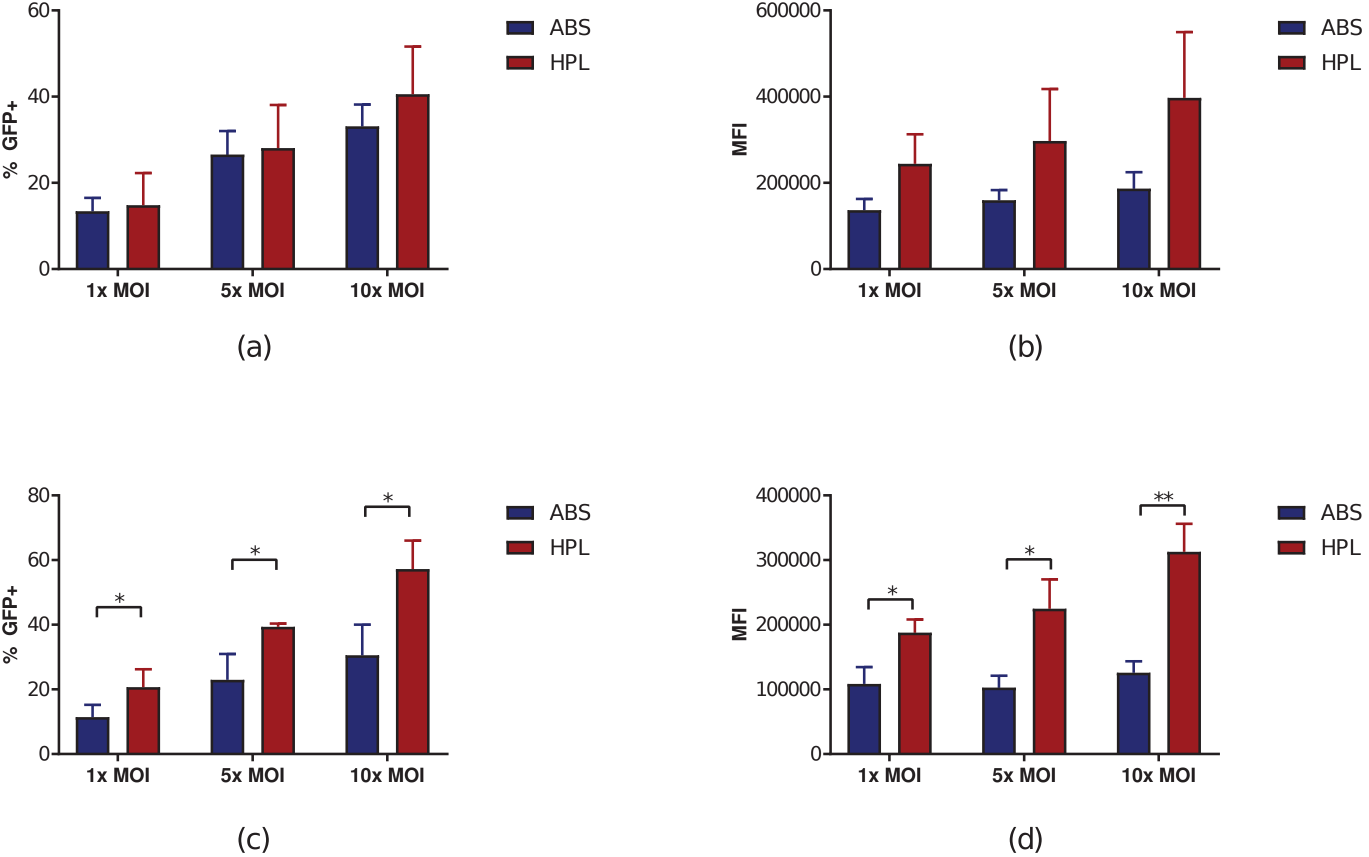

60
40
20
0
% GFP+
ABS
HPL
1x MOI
5x MOI
10x MOI
(a)
600000
400000
200000
0
MFI
ABS
HPL
1x MOI
5x MOI
10x MOI
(b)
80
60
40
20
0
% GFP+
*
*
*
ABS
HPL
1x MOI
5x MOI
10x MOI
(c)
400000
300000
200000
100000
0
MFI
*
*
**
ABS
HPL
1x MOI
5x MOI
10x MOI
(d)

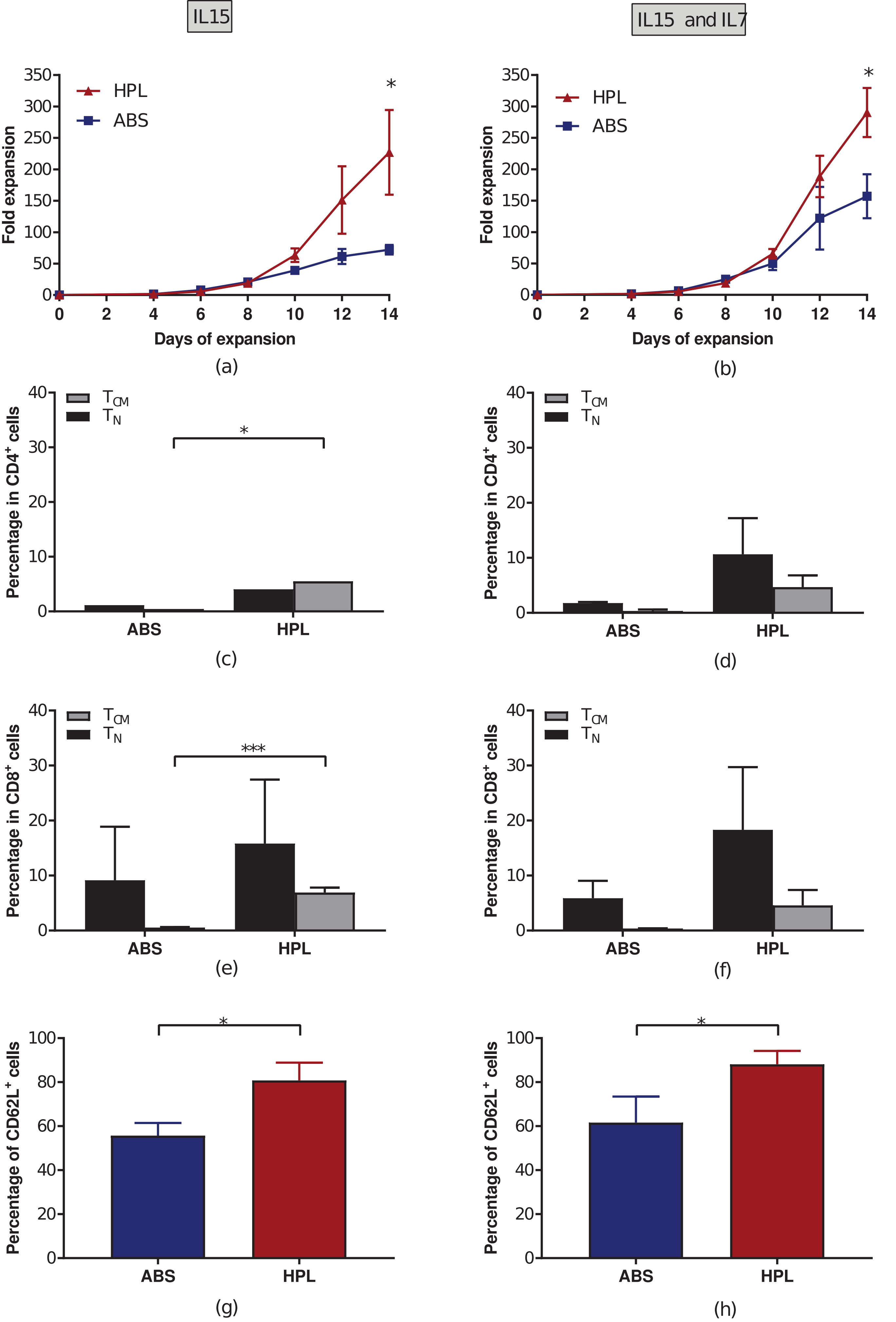

IL15
IL15 and IL7
HPL
ABS
Fold expansion
Days of expansion
(a)
(b)
T_{CM}
T_N
Percentage in CD4+ cells
(c)
(d)
Percentage in CD8+ cells
(e)
(f)
Percentage of CD62L+ cells
(g)
(h)

Supplementary Table S1. Differentially enriched proteins in HPL vs ABS with P ≤ 0.01 and Fold Change ≥ 10

Uniprot_ID	Protein name	P-value	Log of Ratio
P09341	GROa	0.0006730	3.483
P78504	Jagged 1	0.0010730	3.260
P05113	IL-5	0.0002210	3.163
P18510	IL-1ra	0.0018030	3.125
P80162	GCP-2	0.0000370	2.944
P09104	NSE	0.0000010	2.630
Q9Y624	JAM-A	0.0000480	2.318
Q92583	TARC	0.0001370	2.208
P09238	MMP-10	0.0008270	2.192
P01579	IFNg	0.0002220	2.156
P05112	IL-4	0.0000180	2.089
P60568	IL-2	0.0002460	2.005
P08581	HGF R	0.0000140	1.963
P04141	GM-CSF	0.0000050	1.948
Q9Y336	Siglec-9	0.0001640	1.940
P08887	IL-6R	0.0000010	1.922
P01583	IL-1a	0.0000300	1.906
P14174	MIF	0.0000070	1.903
P13232	IL-7	0.0000290	1.893
Q99616	MCP-4	0.0000260	1.846
Q15389	ANG-1	0.0069300	1.815
Q9BX67	JAM-C	0.0004330	1.625
P19971	PD-ECGF	0.0001270	1.624
P35225	IL-13	0.0005850	1.597
Q14112	Nidogen-2	0.0000040	1.591
P42830	ENA-78	0.0000170	1.567
Q9NRJ3	CCL28	0.0054820	1.561
P51671	Eotaxin	0.0013100	1.550
Q96AP7	ESAM	0.0062370	1.536
P07585	Decorin	0.0000140	1.518
P23560	BDNF	0.0007700	1.461
P50895	BCAM	0.0000080	1.449
O43927	BLC	0.0038960	1.445
P22301	IL-10	0.0051650	1.425
Q14126	Desmoglein 2	0.0017920	1.401
P15289	Arylsulfatase A	0.0001780	1.393
P0DJD7/P0DJD8/P0DJD9	Pepsinogen I	0.0002100	1.387
Q9ULL4	Plexin B3	0.0000390	1.380
O43464	HTRA2	0.0034850	1.371
Q96LA5	FCRL2	0.0083850	1.356
P01133	EGF	0.0005820	1.355
P00797	Renin	0.0000090	1.351
P21860	ErbB3	0.0001560	1.348

Q9UHL4	DPPII	0.0000080	1.311
P03956	MMP-1	0.0002740	1.290
O14773	TPP1	0.0000230	1.282
P48551	IFNab R2	0.0006970	1.255
P21741	Midkine	0.0000920	1.247
Q16552	IL-17	0.0003030	1.245
P41159	Leptin	0.0005550	1.224
Q9NRA1	PDGF-CC	0.0005310	1.204
Q14116	IL-18	0.0003830	1.192
Q9NZ53	Endoglycan	0.0023750	1.180
Q14005	IL-16	0.0002400	1.152
Q9Y4D7	Plexin D1	0.0000900	1.147
P10646	TFPI	0.0000420	1.144
P01137	TGFb1	0.0010630	1.115
Q96LC7	Siglec-10	0.0000320	1.082
P01137.2	LAP(TGFb1)	0.0000490	1.046
P30530	Axl	0.0003350	1.042
Q99538	Legumain	0.0000460	1.039
O43508	TWEAK	0.0006400	1.031
P40189	gp130	0.0007550	1.022
P04626	ErbB2	0.0015660	1.016
P04637	p53	0.0005690	1.008
P01127	PDGF-BB	0.0001890	1.006
P78380	LOX-1	0.0087360	-1.697
P46531	Notch-1	0.0024410	-2.843
O14788	TRANCE	0.0001130	-4.781

The differentially enriched proteins were identified with multiple T-test analysis ($P \leq 0.01$) and HPL/ABS ratio of concentrations (fold change ≥ 10).
